# The Rotavirus Surveillance System in Yemen: Evaluation Study

**DOI:** 10.2196/27625

**Published:** 2021-06-08

**Authors:** Eman Abdullah Lardi, Sharaf Sharaf Al Kuhlani, Mohammed Abdullah Al Amad, Abdulwahed Abduljabar Al Serouri, Yousef Saleh Khader

**Affiliations:** 1 Field Epidemiology Training Program Ministry of Public Health and Population Sana'a Yemen; 2 Rotavirus Surveillance Program Ministry of Public Health and Population Sana’a Yemen; 3 Department of Community Medicine, Public Health and Family Medicine Faculty of Medicine Jordan University of Science & Technology Irbid Jordan

**Keywords:** Rotavirus, surveillance system, evaluation, Yemen

## Abstract

**Background:**

Rotavirus (RV) kills over 185,000 children <5 years every year and is responsible for over one-third of all child diarrheal deaths worldwide. The Rotavirus Surveillance System (RVSS) in Yemen was launched in 2007 at five sentinel sites to monitor the impact of the vaccine on RV morbidity and mortality.

**Objective:**

This study aimed to determine the usefulness of the RVSS, assess its performance, and identify the strengths and weaknesses of its implementation.

**Methods:**

The Centers for Disease Control and Prevention’s updated guidelines on evaluating a public health surveillance system were used to evaluate the RVSS. In this assessment, qualitative indicators, such as usefulness, flexibility, stability, simplicity, and acceptability, were assessed through in-depth interviews with stakeholders at the central level and semistructured questionnaires with the sentinel site coordinators. The indicators for quantitative attributes—sensitivity, positive predictive value (PPV), completeness, and timeliness—were assessed by reviewing the results of laboratory samples and a random sample of case report forms. The scores for the indicators were expressed as poor (<60%), average (60% to <80%), and good (≥80%).

**Results:**

The overall usefulness score of the RVSS was 73%, indicating an average rank. The RVSS was rated as having good flexibility (91%) and stability (81%), and average simplicity (77%) and acceptability (76%). In terms of quantitative attributes, the system was poor for sensitivity (16%), average for PPV (73%), and good for completeness (100%) and timeliness (100%).

**Conclusions:**

Although the system attributes were flexible, stable, capable of providing quality data, and performing timely data reporting, some attributes still needed improvements (eg, usefulness, simplicity, acceptability, and PPV). There is a need for a gradual replacement of donor funds with government funds to ensure sustainability. The RVSS in Yemen strongly requires a progressive increase in the number of sites in governorates and sensitivity enhancement.

## Introduction

Rotavirus (RV) is the major cause of vaccine-preventable severe and fatal diarrhea among young children [1]. Infection can be asymptomatic, cause mild to moderate gastroenteritis, or severe gastroenteritis with dehydration requiring hospitalization [2]. Recovery from a first RV infection usually does not lead to permanent immunity, and reinfection can occur at any age but with less severity than the first. The World Health Organization (WHO) identified an RV vaccine as the key strategy in reducing the RV-diarrhea burden. The monovalent (RV1) Rotarix and the pentavalent (RV5) Rota Teg are two safe and effective oral vaccines against RV infection in children [3].

Globally, nearly every child in the world gets infected with RV between 3 and 5 years of age. However, the highest rates of severe disease occur commonly at the age of 6 to 24 months [3,4]. Studies in the Eastern Mediterranean Region (EMR) have estimated approximately 65,000 child deaths each year due to RV infection. Mortality remains high in this region, especially in countries with a lower per capita income, such as Pakistan, Afghanistan, Sudan, Yemen, and Somalia [5]. The countries with a higher per capita income have few deaths, but the burden of severe RV disease is reflected in the many hospitalizations and clinic visits among children <5 years of age [5].

Yemen is a resource-limited country with acute gastroenteritis–related morbidity and mortality as the major health problem. The Ministry of Public Health and Population (MoPH&P) had introduced the RV vaccine into the routine immunization schedule in 2012. The vaccine is administered in two doses: the first dose is administrated at 6 weeks of age, and the second dose is completed by 10 weeks of age [6]. The introduction of the RV vaccine helped to decrease the burden of severe RV gastroenteritis and RV-associated mortality [7]. The RV hospitalization incidences in Yemen decreased from 43.8% in 2009 to 10.5% in 2014 [7].

The Rotavirus Surveillance System (RVSS) was launched in 2007 at five sentinel sites to monitor the impact of the vaccine on RV morbidity and mortality. The RVSS has never been evaluated before in Yemen. Therefore, this study aimed to determine the usefulness and performance of the RVSS, and identify the strengths and weaknesses of the system implementation.

## Methods

### Evaluation Design

The Centers for Disease Control and Prevention’s (CDC) updated guidelines on evaluating a public health surveillance system were used to evaluate the RVSS [8]. In this assessment, qualitative indicators of usefulness and other attributes of the system (eg, flexibility, stability, simplicity, and acceptability) were assessed through in-depth interviews with stakeholders at the central level, and semistructured questionnaires were used with the sentinel site coordinators. Furthermore, the indicators for quantitative attributes such as sensitivity, positive predictive value (PPV), completeness, and timeliness were assessed by reviewing the results of laboratory samples and a random sample review of case report forms. All the five sentinel sites covered by the RVSS (Yemen Swedish Hospital in Taiz, Al Wahda General Teaching Hospital in Aden, Al Sabeen Maternal Hospital in Sana’a, Al-Thawra Hospital in Ibb, and Al-Thawra Hospital in Al Hudaydah) were included and established for qualitative evaluation. The RVSS evaluation was conducted from October to December 2018.

### Evaluation Approach

The RVSS stakeholders at the central level and the sentinel site coordinators were included in this study. Different data collection methods were used, such as a desk review of the RVSS documents, in-depth interviews with stakeholders at the central level, and semistructured questionnaires with the sentinel site coordinators. The evaluation involved reviewing the available documents such as operational manuals, monthly and annual reports, and databases. The documents were reviewed before interviewing the stakeholders to obtain information about the RVSS. Seven in-depth interviews were conducted with the stakeholders at the central levels to understand the RVSS implementation, as well as its usefulness, flexibility, stability, and strengths and weaknesses. The indicators for usefulness and other qualitative attributes were developed according to the CDC guidelines. A registers review was used to assess the quantitative attributes (sensitivity, PPV, completeness, and timeliness).

The indicators of attributes (usefulness, flexibility, and stability of the system) were assessed using questions with “yes” or “no” answers that were scored as 1 or 0, respectively. The level of simplicity and acceptability of the system was assessed on a 5-point Likert scale (1=strongly disagree, 2=disagree, 3=neutral, 4=agree, and 5=strongly agree).

For each indicator, the score percent was calculated as:


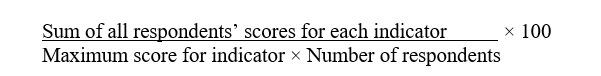


The overall attribute score percent was calculated as:


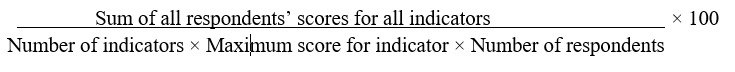


The sensitivity of the system was assessed by the proportion of stool samples of the suspected cases of RV gastroenteritis that tested positive for RV. The PPV was calculated as the proportion of the positive RV stool samples reported by the sentinel sites that tested positive at the National Central Public Health Laboratory (NCPHL). Timeliness was measured as the proportion of reports sent to the central level by the deadline. Missing data were measured by selecting the 1-year data and calculating the percentages of the missed variables. The data accuracy was assessed by comparing reports at the central level with the case report form. The ranking and scoring system used for the quantitative and qualitative attributes, as well as for the indicators of each attribute, were as follows: poor (<60%) average (60% to <80%), and good (≥80%).

### Ethical Approval

The ethical review committee of MoPH&P advised that ethical approval for this evaluation protocol was not needed as it was part of the ongoing national evaluation activity. The stakeholders at the central level and the sentinel site coordinators were explained the aim of the study and were requested to participate. If they agreed, either an interview was conducted or a semistructured questionnaire was administered. Confidentiality of the collected data was maintained by limiting access to the research team only.

## Results

### Description of the RVSS

The MoPH&P established the RVSS in 2007 with technical and financial support from the WHO. After the RV vaccines were introduced in Yemen in 2012, the objectives of the RVSS were updated to include assessments of the vaccine impact on RV morbidity and mortality among children <5 years, as well as changes in RV epidemiology and circulating strains, and provide a basis for further epidemiologic research.

**Figure 1:**
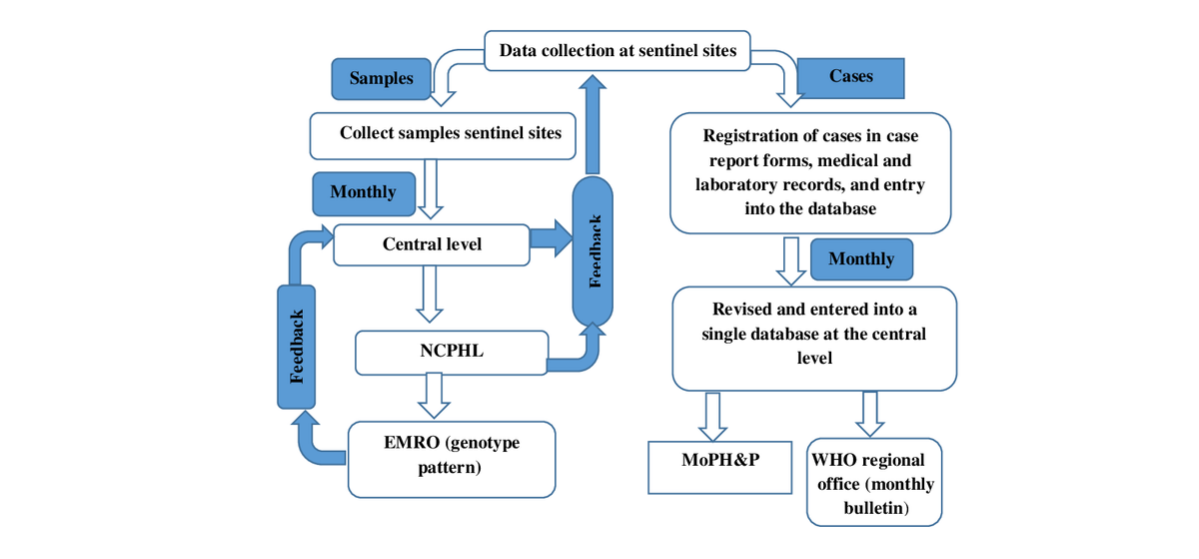
Flow chart for RVSS data collection methods employed in phases 1 and 2. CDC: Centers for Disease Control and Prevention, EMRO: Regional Office for the Eastern Mediterranean, MoPH&P: Ministry of Public Health and Population, NCPHL: National Central Public Health Laboratory, RVSS: Rotavirus Surveillance System, WHO: World Health Organization.

The RVSS required collecting data on individual cases of diarrhea among children <5 years of age. It was the active surveillance at the five sentinel sites. The potential data sources included inpatients in the Department of Pediatrics, the Emergency Department, and the Diarrhea Treatment Centers. A suspected case was a case with an acute (<14 days) watery diarrhea, defined as 3 or more loose or watery stools within a 24-hour period in a child <5 years of age admitted for diarrhea treatment into the hospital ward or the emergency unit at the sentinel sites. Children with bloody diarrhea and nosocomial infections were excluded. A confirmed case was a suspected case with the presence of RV in its stool confirmed by an enzyme immunoassay (EIA) or polymerase chain reaction (PCR)–based methods. Figure 1 shows the RVSS data collection flow chart.

### In-depth Interviews With Stakeholders at the Central Level

#### Usefulness

Table 1 highlights that 5 out of 8 usefulness indicators achieved a good rank, while 2 indicators, development of the national policy strategy for the national immunization program and estimation of the RV magnitude, incidence, and mortality, achieved a poor rank. Another indicator, planning the resources, achieved an average rank. The overall usefulness indicated an average rank (n=41, 73%).

**Table 1:** The scores (score percent, %) and rank of the usefulness indicators as assessed by the central-level stakeholders.

Indicator	Score (%)	Rank
The system data provide an estimate of rotavirus magnitude, incidence, and mortality	2 (29)	Poor
The system data detect trends of rotavirus spread over time	7 (100)	Good
The system data recognize high-risk groups	7 (100)	Good
The system data plan the resources for prevention and control	5 (71)	Average
The system data update and develop the national policy strategy for the national immunization program	2 (29)	Poor
The system data assess the effect of interventions	6 (86)	Good
The system data estimate the needs of laboratory kits	6 (86)	Good
The system data are used as the basis for epidemiologic research	6 (86)	Good
Overall usefulness	41 (73)	Average

#### Flexibility

Table 2 shows that 5 out of 6 flexibility indicators achieved a good rank, while the indicator, “The system can accommodate any changes in funding,” was ranked poor. The overall flexibility achieved a good rank (n=38, 91%).

**Table 2:** The scores (score percent, %) and rank of the flexibility indicators as assessed by the central-level stakeholders.

Indicator	Score (%)	Rank
The system can accommodate changes in the number of sentinel sites	7 (100)	Good
The system can accommodate any changes in funding	4 (57)	Poor
The system can accommodate changes in case definition	7 (100)	Good
The system can accommodate changes in reporting method	7 (100)	Good
The system can be adapted to integrate with other surveillance systems	6 (86)	Good
The system can accommodate changes in data with minimum cost and efforts	7 (100)	Good
Overall flexibility	38 (91)	Good

#### Stability

The study showed that 4 out of the 6 stability indicators achieved a good rank (Table 3). However, 2 indicators, the availability of planned resources for maintenance and the sustainability of the system if donors withdrew their support, achieved a poor rank. The overall stability ranked good (n=34, 81%).

**Table 3:** The scores (score percent, %) and rank of the stability indicators as assessed by the central-level stakeholders.

Indicator	Score (%)	Rank
No unscheduled system outages occurred during the last month	7 (100)	Good
No electrical power outage occurred during the last week	6 (86)	Good
There are planned resources for the maintenance of the system	4 (57)	Poor
The system is stable even after the sponsor’s withdrawal of support	3 (43)	Poor
The system does not require time to manage data	7 (100)	Good
Reports are released regularly	7 (100)	Good
Overall stability	34 (81)	Good

#### Strengths and Weaknesses

The majority of stakeholders (n=6, 86%) reported the presence of a qualified medical team at the central and terminal levels as one of the strengths of the RVSS. Around half (n=4, 57%) respondents said the accuracy of data as an important strength:

Another strength was the continuity of data flow to the central level.

Regarding the weaknesses in the RVSS, all stakeholders said that a lack of financial support from the government and total dependence on the WHO for support were the major weaknesses of the system. The small number of surveillance sites was another reported weakness. One participant said:

Other weaknesses included delays in sending the feedback about the samples’ results by the WHO Regional Office for the Eastern Mediterranean (WHO EMRO), lack of laboratory kits, and poor utilization of research findings*.*

### Semistructured Questionnaire With the Sentinel Site Coordinators

The five sentinel site coordinators (all were females) responded to the semistructured questionnaire.

#### Simplicity

Table 4 shows that 8 out of 10 simplicity indicators (eg, case definition is available and easy to use, less time spent on collecting data, etc) achieved a good rank, while 2 indicators, the availability of laboratory tests and training, achieved a poor rank, and the overall simplicity was ranked average (n=193, 77%).

**Table 4:** The scores (score percent, %) and rank of the simplicity indicators as assessed by the sentinel site coordinators.

Indicator	Score (%)	Rank
The rotavirus case definition is available	25 (100)	Good
The rotavirus case definition is easy to use	24 (96)	Good
The case report form is available	24 (96)	Good
The case report form is easy to use	24 (96)	Good
Less time spent on collecting data	20 (80)	Good
Transmitting data to the enteral level is easy	22 (88)	Good
Follow-up of cases is easy	20 (80)	Good
Laboratory tests available in the health facility to confirm a diagnosis	5 (20)	Poor
You received training for rotavirus surveillance	22 (88)	Good
Training courses are conducted frequently	7 (28)	Poor
Overall simplicity	193 (77)	Average

#### Acceptability

Table 5 displays the 4 acceptability indicators used in the study. It was found that the indicators willingness to participate in the RVSS and responsiveness of the system to suggestions achieved a good rank. However, satisfaction with the RVSS and receiving feedback from the central level achieved average and poor ranks, respectively. The overall acceptability achieved an average rank (n=76, 76%).

**Table 5:** The scores (score percent, %) and rank of the acceptability indicators as assessed by the sentinel site coordinators.

Indicator	Score (%)	Rank
You are willing to participate in the Rotavirus Surveillance System	24 (96)	Good
You are satisfied with the surveillance system	17 (68)	Average
Received feedback report from the central level	12 (48)	Poor
Responsiveness of the system to suggestions	23 (92)	Good
Overall acceptability	76 (76)	Average

### Assessment of Quantitative Attributes: Sensitivity, PPV, Completeness, and Timeliness

Of the 1787 cases suspected of having RV gastroenteritis at the sentinel sites, 1542 had their stool samples tested. Of the total cases tested for the stool samples, only 16% (n=244) samples tested positive for RV, indicating poor sensitivity. However, for PPV, about 73% (n=178) of the positive RV stool samples reported by the sentinel site tested positive at the NCPHL.

There were no missing variables when a random sample of 30 case report forms was reviewed. These forms were found to be consistent with the database. Therefore, completeness was 100% (n=30). All sentinel sites sent their reports by the fifth day of next month. Therefore, timeliness was also 100% (n=5).

### Overall Performance of the RVSS

The overall RVSS performance was found to be average (Table 6).

**Table 6:** Summary of the overall performance of the Rotavirus Surveillance System (RVSS).

Attributes	Score (%)	Rank
Performance according to the central level	113 (81)	Good
Performance according to sentinel sites	269 (77)	Average
Performance of quantitative attributes	215 (57)	Poor
Overall RVSS performance	597 (69)	Average

## Discussion

### Principal Findings

The RVSS performance evaluation could enhance the usefulness of the surveillance data for public health action. In this evaluation, we assessed the attributes and operation of the RVSS in Yemen using the CDC’s updated guidelines [8]. The RVSS data helped estimate the RV severity and provided a basis for epidemiologic research. However, it was reported that the RVSS data were used poorly to update and develop the national policy strategy for the national immunization program in Yemen. In contrast, the Australian Rotavirus Serotyping Program evaluation showed good usefulness of the system [9].

The flexibility of the RVSS was rated as good, and the system appeared to be able to adapt and accommodate new changes such as changes in the RV reporting method (phone reporting) and changes in the case definition. However, the system was found to be considerably donor dependent and could poorly accommodate any changes in funding. These observations were different from the evaluation results of the Australian Rotavirus Serotyping Program, which showed the system to be flexible and able to adapt to the changes [9]. Similarly, the RVSS evaluation in Kenya demonstrated the system to be flexible as it could incorporate new reporting sources [10].

The stability of the RVSS was rated good in this study. Although the system was stable and did not require time to manage the data, the system was considered poorly stable if the donors withdrew their support. The RVSS simplicity was rated average, while the case definition and the surveillance case report forms were reported to be available and easy to use. However, the laboratory tests in the health facility were not available to confirm a diagnosis. A comparison with the systems available in other countries showed that the Kenya RVSS scored better on simplicity [10].

The acceptability of the RVSS was rated as average, reflected by the stakeholders’ willingness to participate in the RVSS and the responsiveness of the system to suggestions. However, the stakeholders’ satisfaction with the RVSS and receiving feedback from the central level achieved average and poor rankings, respectively. For the last 2 years, the system did not receive any feedback reports from the WHO EMRO laboratories.

The sensitivity of the system was poor as only 16% of stool samples tested positive, which was contrary to a previous evaluation from Australia that found the system sensitive [9]. Moreover, the PPV in this evaluation was 73%, whereas a PPV of 98.5% was reported from the Kenyan RVSS evaluation [10]. Completeness was 100%, which was slightly higher than the results reported in the Kenya RVSS evaluation, where the completeness was 88% [10].

Our evaluation had several limitations. The assessment did not include one of the essential attributes of the system, representativeness, because the system depended on only five sentinel sites in five governorates and did not include the rest of the governorates or health facilities. Furthermore, we could not assess the timeliness regarding the feedback on the samples from the WHO EMRO during the period 2017-2018 because the WHO stopped receiving samples since 2017. Regarding the sensitivity and PPV, our evaluation was based only on the results of the 2017-tested samples. The sensitivity of the 2018 samples was not evaluated because the samples were not tested due to a lack of laboratory kits.

### Conclusions

Although the system attributes were flexible, stable, capable of providing quality data, and performing timely data reporting, some attributes still needed improvement (eg, usefulness, simplicity, acceptability, and PPV). The system sustainability requires planning a gradual replacement of donor funds with government funds. Additionally, it is imperative that the NCPHL be upgraded with a RV genotype testing facility and has a scaled-up RVSS with more sites in governorates. There is a greater need for sensitivity enhancement of the RVSS. There is a need to ensure timely feedback from the WHO EMRO on the results of samples. Regular refresher training and feedback for health staff at the sentinel sites are recommended.

## Abbreviations

CDC: Centers for Disease Control and Prevention
EIA: enzyme immunoassay
EMR: Eastern Mediterranean Region
MoPH&P: Ministry of Public Health and Population
NCPHL: National Central Public Health Laboratory
PCR: polymerase chain reaction
PPV: positive predictive value
RV: Rotavirus
RVSS: Rotavirus Surveillance System
WHO: World Health Organization
WHO EMRO: WHO Regional Office for the Eastern Mediterranean
